# Pachymic Acid Targets PI3K/Akt Signaling Pathway to Attenuate tPA‐Induced Hemorrhagic Transformation After Ischemic Stroke

**DOI:** 10.1002/cns.70782

**Published:** 2026-02-13

**Authors:** Yongshi Wu, Tong Zhang, Ruoqi Li, Yige Wu, Congmin Wei, Fangming Sun, Shanshan Zhang, Xiang Fan

**Affiliations:** ^1^ School of Basic Medical Sciences Zhejiang Chinese Medical University Hangzhou China

**Keywords:** blood–brain barrier, hemorrhagic transformation, ischemic stroke, Pachymic acid, PI3K/Akt signaling pathway, tPA

## Abstract

**Background:**

Tissue plasminogen activator (tPA)–induced cerebral hemorrhagic transformation (HT) after ischemic stroke limits its clinical use widely. Pachymic acid, a main active component of *Poria cocos*, mitigates brain ischemia/reperfusion injury, but its effect on tPA‐induced HT is unclear.

**Methods:**

A focal middle cerebral artery occlusion/reperfusion model was established and administered with tPA and pachymic acid. Infarct volume and neurological function were assessed at 24 h after reperfusion. Blood–brain barrier (BBB) damage was evaluated using Evans blue leakage, immunofluorescence, and Western blot. Pachymic acid and PI3K protein interaction was identified using molecular docking, molecular dynamics (MD) simulation, and surface plasmon resonance (SPR).

**Results:**

Compared with the tPA group, pachymic acid dose‐dependently improves neurological and motor functions, reduces infarct volume and hemorrhagic volume, and alleviates permeability and tight junction protein degradation of BBB after ischemic stroke, with the strongest effects observed at the highest dose. Molecular docking, MD simulation, and SPR results indicate that pachymic acid can directly bind to PI3K protein. Further experiments showed that the PI3K inhibitor LY294002 reversed pachymic acid's protective effects.

**Conclusions:**

This study demonstrated that pachymic acid protects the BBB by targeting PI3K to activate the PI3K/Akt signaling pathway, thereby alleviating tPA‐induced HT after ischemic stroke.

## Graphical abstract

Pachymic acid protects blood–brain barrier (BBB) integrity by decreasing the degradation of tight junction proteins caused by tissue plasminogen activator (tPA). Pachymic acid activates the PI3K/Akt pathway via direct binding to PI3K protein, mitigating BBB damage and hemorrhagic transformation induced by tPA after ischemic stroke.

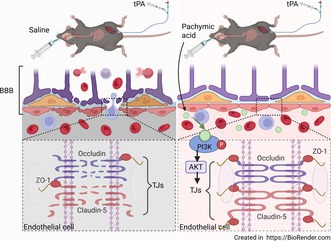

**Abbreviations**
CCA: common carotid artery
ECA: external carotid artery
HT: hemorrhagic transformation
I/R: ischemia/reperfusion
ICA: internal carotid artery
MCAO/R: middle cerebral artery occlusion/reperfusion
MD: Molecular dynamics
MMP‐9: matrix metalloproteinase‐9
SPR: Surface plasmon resonance
tPA: tissue‐type plasminogen activator
ZO‐1: Zonula occludens‐1

## 1 Introduction

Stroke stands as a leading global cause of mortality and disability [1]. Current management of acute ischemic stroke relies on reperfusion strategies, primarily intravenous thrombolysis and mechanical thrombectomy. Tissue‐type plasminogen activator (tPA) and the newer generation thrombolytic agent, tenecteplase (TNK), are key pharmacological options [2, 3, 4]. Despite the emergence of novel agents and techniques, tPA, as the classic thrombolytic approved by the U.S. Food and Drug Administration (FDA) for acute ischemic stroke, remains at the core of investigative efforts. Although successful clinical trials have extended the therapeutic time window for intravenous tPA thrombolysis from 3 to 4.5 h [5], this is accompanied by a significantly increased risk of hemorrhagic transformation (HT). Thus, minimizing tPA‐induced HT after ischemic stroke is an urgent clinical challenge that will benefit more patients.

The increase in blood–brain barrier (BBB) permeability induced by tPA is one of the main causes of HT. Studies have shown that tPA promotes the degranulation of neutrophils and the release of matrix metalloproteinase‐9 (MMP‐9). MMP‐9 can degrade the extracellular matrix of brain microvascular endothelial cells, thereby increasing BBB permeability and promoting HT. And published literature already demonstrates that suppressing the NF‐κB/MMP‐9 inflammatory axis effectively alleviates tPA‐induced BBB disruption and hemorrhage [6]. Therefore, BBB permeability is one of the therapeutic strategies to improve tPA‐induced HT.

The PI3K/Akt is an essential signaling pathway that regulates cell growth and differentiation. Its activation post‐cerebral ischemia–reperfusion (I/R) injury alleviates oxidative stress, inhibits inflammatory responses, reduces neuronal apoptosis, autophagy, and pyroptosis, and promotes neurological recovery. Additionally, it mitigates increased BBB permeability and brain hemorrhage post‐stroke, significantly reducing hemoglobin content [7]. These findings suggest that the PI3K/Akt pathway is a promising therapeutic target for lowering tPA‐induced HT after ischemic stroke [8]. Therefore, developing effective PI3K/Akt activators may be a future research focus to mitigate tPA‐induced HT after ischemic stroke.

*Poria cocos*, a traditional medicinal and edible material, has diuretic, spleen‐strengthening, and sedative effects, and exhibits antioxidant and anti‐inflammatory properties that can mitigate oxidative stress and inflammatory damage to the BBB. Research indicates that *Poria cocos* extracts promote microglial polarization from the pro‐inflammatory M1 to the anti‐inflammatory M2 phenotype via the IRF5‐IRF4 axis, reducing inflammation and preventing tPA‐induced HT [9]. Pachymic acid is an important triterpenoid compound in *Poria cocos* and one of its main active ingredients. Studies have shown that pachymic acid can activate the PI3K/Akt pathway, significantly upregulate the expression of BBB‐related proteins, such as tight junction proteins ZO‐1, VE‐cadherin, and Occludin, reduce neuronal damage after I/R in rats, and reduce neuronal apoptosis [10], and pachymic acid can cross the BBB efficiently [11]. However, the potential of pachymic acid to reduce HT induced by tPA has not been fully explored. Therefore, this study further investigated whether pachymic acid can reduce HT induced by tPA through BBB protection by establishing a middle cerebral artery occlusion/reperfusion (MCAO/R) animal model and explored its possible mechanisms.

## 2 Materials and Methods

### 2.1 Experimental Animals

Male C57BL/6J mice (SPF), weighing 21–24 g (6–8 weeks old), were provided by Zhejiang Vital River Laboratory Animal Technology Co. Ltd. Mice were housed at 25°C ± 1°C and 50%–60% humidity, with free access to food and water under a 12 h light–dark cycle.

### 2.2 MCAO/R Models Establishment

C57BL/6J mice were fasted for 12 h, then anesthetized with 2.5% isoflurane and maintained with 1% isoflurane, 30% oxygen, and 70% nitrous oxide. MCAO/R model was established based on our previous study [12]. Briefly, an incision was made in the neck of the mouse to isolate the left common carotid artery (CCA), internal carotid artery (ICA), and external carotid artery (ECA). The CCA and ICA were closed by arterial clips, and after arteriotomy in the ECA, a silicone‐coated 6–0 monofilament (0.20 ± 0.01 mm) was advanced through the ICA toward the middle cerebral artery at the incision. After modeling, the incision was sutured and disinfected. Rectal temperature was maintained at 36.5°C–37°C using an electric heating pad during and after surgery. Food and water were provided postoperatively. Cerebral blood flow was monitored using a MoorVMS‐LDF2 (Moor Instruments, USA), with successful occlusion defined as a > 70% reduction in blood flow. Reperfusion was achieved by removing the monofilament after 1 h of ischemia.

### 2.3 Experimental Groups and Pachymic Acid Administration

The monofilament was removed to restore blood flow after 1 h of ischemia, and the left femoral vein was cannulated for tPA administration. tPA (Actilyse; Boehringer Ingelheim) was dissolved in sterile water and administered intravenously at a dose of 10 mg/kg [13] (10% of the dose was injected over 1 min, and the remaining 90% was injected over 30 min) using a syringe infusion pump after reperfusion. For pharmacodynamic studies, mice were randomly assigned to Sham, MCAO, tPA + Vehicle, tPA + pachymic acid‐20 mg/kg, tPA + pachymic acid‐40 mg/kg, and tPA + pachymic acid‐80 mg/kg groups. For mechanistic studies, mice were divided into MCAO, tPA + Vehicle, tPA + pachymic acid‐80 mg/kg, and tPA + pachymic acid‐80 mg/kg + LY294002 groups. Pachymic acid (lemeitian; 29,070–92‐6) was dissolved with 5% DMSO in saline and administered by oral gavage during reperfusion. Sham, MCAO, tPA + Vehicle group received oral gavage with the same volume and formulation of 5% DMSO in saline.

### 2.4 Neurobehavioral Evaluation

mNSS: At 24 h after reperfusion, a blinded researcher assessed neurological impairment in mice using the mNSS test [14]. Higher scores indicate more severe neurological deficits.

Foot‐fault test [15]: This test evaluated paw placement accuracy on a 40 × 40 × 50 cm metal grid with 2 × 2 cm openings. A foot‐fault was recorded when a paw slipped and fell from the grid during a 60 s trial. Data are presented as the proportion of foot‐faults relative to total steps.

Adhesive removal test [16]: This test was used to assess sensory‐motor deficits in the forepaws of mice. Mice underwent habituation training before the experiment. Tapes were affixed to the forepaws, and the time taken to first touch and remove the tape was recorded within 120 s. Data from the contralateral impaired forepaws were analyzed.

### 2.5 2,3,5‐Triphenyltetrazolium Chloride (TTC) Staining

Seven coronal slices with a thickness of 1 mm were taken from the brain. 1.5% TTC was used to dye the slices. ImageJ software (NIH) was used to analyze the infarct volume. The proportion of infarct was analyzed using the formula below: Infarct volume (%) = (Contralateral volume–Ipsilateral nonischemic volume) × 100/Contralateral volume.

### 2.6 Hemorrhage Transformation Score

Brain tissue was sectioned coronally from the frontal to the occipital pole at 1 mm thickness, resulting in 7 slices. These slices were placed in a flat dish, photographed, and scored for hemorrhage based on the following criteria: non‐hemorrhagic (score 0), small petechiae (score 1), more fused petechiae within the injury area (score 2), blood occupying < 30% of the infarct focus (score 3), and blood occupying > 30% of the infarct focus (score 4) [17].

### 2.7 Assessment of HT

The brain was rapidly harvested and homogenized in 1 mL of ice‐cold PBS, then centrifuged at 13,000 g for 30 min. The supernatant was transferred to a sterile enzyme‐free tube containing Drabkin's reagent and incubated at 37°C for 15 min. The optical density of the sample was measured by spectrophotometry at 540 nm, and hemorrhage volumes were determined using a standard curve.

### 2.8 Evans Blue Leakage Test

After 24 h of reperfusion, mice were injected with 2% Evans Blue (4 mL/kg) via the tail vein. After 2 h of circulation, mice were anesthetized and subjected to cardiac perfusion. The ischemic hemisphere was removed, weighed, homogenized in 10 μL/mg Trichloroacetic acid, and centrifuged at 5000 rpm for 10 min at 4°C. The absorbance of the supernatant (100 μL) was measured at 630 nm.

### 2.9 Immunofluorescence Staining

Brain tissues were post‐fixed in 4% paraformaldehyde for 24 h, then dehydrated in a sucrose gradient at 4°C. Coronal sections (20 μm) were cut, permeabilized with 0.15% Triton X‐100 for 15 min, and blocked with 3% donkey serum for 1 h. Sections were blocked with 3% BSA and incubated overnight at 4°C with primary antibodies: CD31 (1:200, GB11063‐3‐100, Servicebio), ZO‐1 (1:250, 339,100, Invitrogen), MMP‐9 (1:200, Ab283575, Abcam), Occludin (1:50, 404,700, Invitrogen), or Claudin‐5 (1:50, 900,900, Invitrogen), then treated with Alexa Fluor 488 (green) (1:300, HA1125, Huabio) and Alexa Fluor 594 (red) (1:300, HA1122, Huabio) secondary antibodies for 1 h at room temperature. Nuclei were counterstained with DAPI. Images were captured using a Zeiss fluorescence inverted microscope (ZEISS Axio Vert. A1), and immunofluorescence intensity was quantified using ImageJ software.

### 2.10 Western Blotting Analysis

Proteins from cortical and peri‐hemorrhagic brain tissues were extracted using RIPA lysis buffer containing phosphatase and protease inhibitors. Protein concentrations were quantified using the BCA protein assay kit and adjusted to 2 μg/μL. Samples were denatured at 100°C for 10 min, separated by SDS‐PAGE, and transferred to PVDF membranes. The membranes were blocked with 5% Blotting Grade and incubated overnight with primary antibodies: ZO‐1 (1:1000, 339,100, Invitrogen), Occludin (1:1000, 404,700, Invitrogen), Claudin‐5 (1:1000, 900,900, Invitrogen), PI3K (1:1000, 4292S, Cell Signaling Technology), p‐PI3K (1:1000, 17,366 T, Cell Signaling Technology), p‐Akt (1:1000, 4060S, Cell Signaling Technology), Akt (1:1000, 9272S, Cell Signaling Technology), β‐tubulin (1:10,000, ET1602‐4, Huabio), and β‐actin (1:10,000, EM21002, Huabio). Secondary antibodies were incubated for 1.5 h, and protein band densities were measured using an Amersham Imager 680.

### 2.11 Intracerebroventricular Injection

Mice were anesthetized with isoflurane and fixed on the stereotactic apparatus. The scalp was disinfected with 75% alcohol and incised, and the periosteum was bluntly separated to expose the bregma and lambda. A hole was drilled 1.8 mm posterior to the bregma and 1.6 mm left of the midline. Using a microinjector, 10 μL of 10 mM LY294002 solution was injected at a depth of 2.0 mm at 1 μL/min, with the needle retained for 10 min. After a 1‐h recovery, mice underwent MCAO/R surgery. The sham group received 10 μL of 0.9% saline via the same method.

### 2.12 Molecular Dynamics (MD) Simulation

MD simulations of protein–ligand complexes were performed using GROMACS 2019.6 [18] to explore receptor–ligand interactions [19]. The amber99sb‐ildn force field and GAFF were used for proteins and ligands, respectively. Each protein atom was positioned > 1.0 nm from the simulation box edge. The box was filled with SPC216 water and neutralized with Na^+^ and Cl^−^ ions. The system was optimized via steepest descent minimization to eliminate unreasonable contacts and atom overlap. Pre‐equilibration was achieved using NVT and NPT ensembles for 100 ps at 300 K and 1 bar, respectively. A 20,000‐ps MD simulation was then performed under periodic boundary conditions, maintaining temperature (300 K) and pressure (1 bar) using the V‐rescale and Parrinello‐Rahman methods, respectively [20]. The leapfrog algorithm with a 2‐fs time step was used for integration. Long‐range electrostatics were calculated using PME with a Fourier spacing of 0.16 nm, and bond lengths were constrained using LINCS. Trajectories were analyzed and visualized using VMD (v1.9.3) and PyMOL (v2.4.1) [21].

### 2.13 Surface Plasmon Resonance (SPR)

Experiments were conducted using a Biacore T100 system. Recombinant human PI3K protein (Sino Biological, P27‐122DH‐50) was immobilized on a CM5 sensor chip. Pachymic acid was dissolved in PBS containing 5% DMSO and injected into the flow cell at 25°C at a flow rate of 10 μL/min at various concentrations (50, 25, 12.5, 6.25, and 3.125 μM). The sensor chip was washed with alkaline electrophoresis buffer between injections. Proteins were immobilized in different channels on the same chip, and response values obtained by injecting blank buffer were used as controls. Kinetic parameters and affinity constants were determined using Biacore T100 evaluation software.

### 2.14 Statistical Analysis

Data from all experimental groups are presented as means ± standard deviation from at least three independent experiments. Statistical analyses were performed using the SPSS Statistics 26.0 software package. Data normality was assessed using the Shapiro–Wilk test. For normally distributed variables, one‐way analysis of variance (ANOVA) followed by Bonferroni post hoc testing was used to evaluate inter‐group differences. For variables that were not normally distributed, the Kruskal‐Wallis test and the Mann–Whitney U test were employed to examine inter‐group differences. *p* < 0.05 was considered to indicate a statistically significant difference.

## 3 Results

### 3.1 Pachymic Acid Ameliorates Cerebral HT and Neurological Deficits Induced by tPA After Ischemic Stroke

**FIGURE 1:**
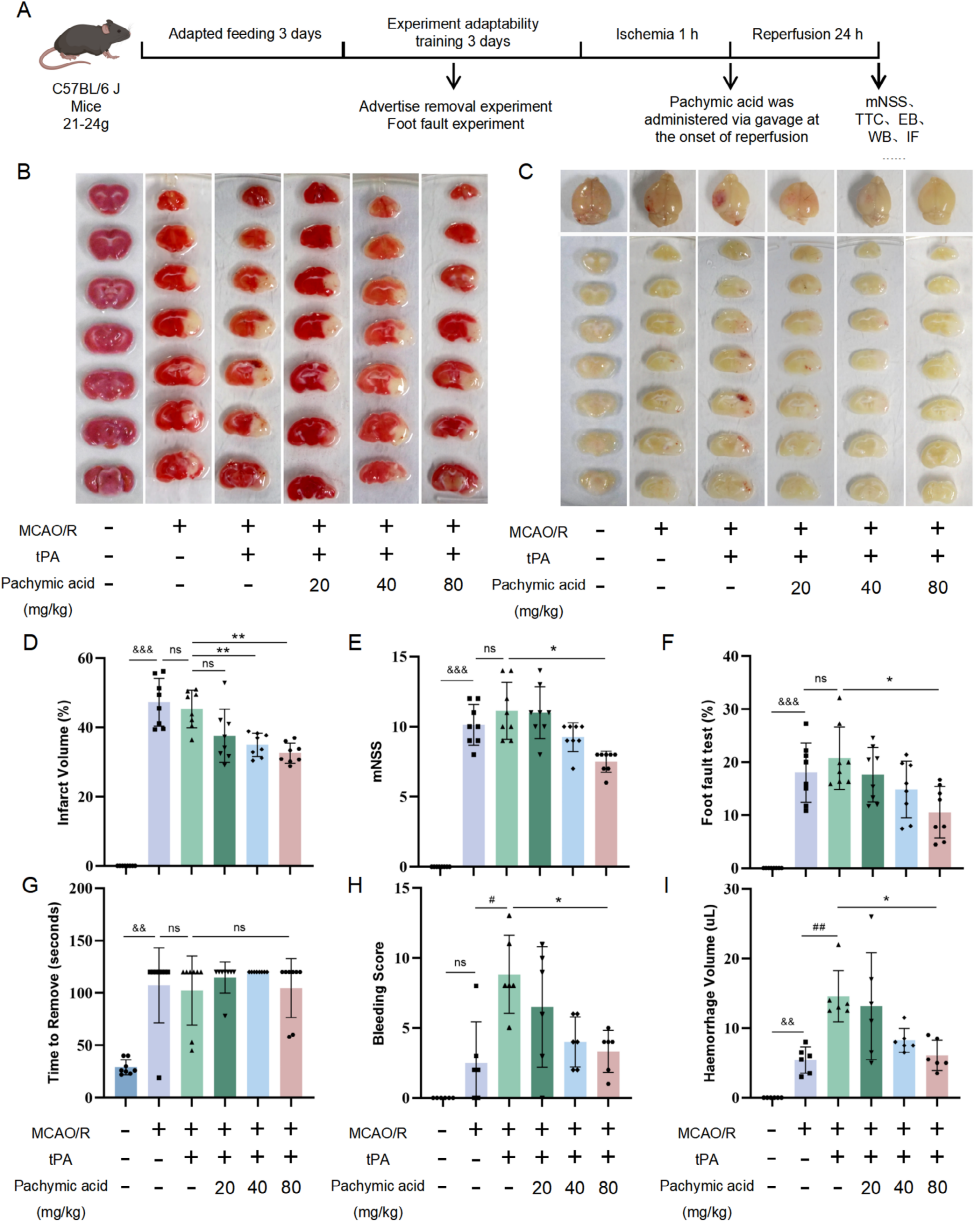
Pachymic acid ameliorates cerebral HT and neurological deficits induced by tPA after ischemic stroke. (A) Timeline of the experimental protocol. (B) Representative images of TTC staining for infarct volume at 24 h after tPA treatment for ischemic stroke. (C) Representative images of brain coronal sections and top views at 24 h after tPA treatment for ischemic stroke. (D) Quantitative analysis of TTC‐stained infarct volume (*n* = 10). The effects of pachymic acid on neurobehavioral deficits in MCAO/R mice, as assessed by mNSS (*n* = 8, E), foot‐fault test (*n* = 8, F), and adhesive removal test (*n* = 8, G). (H) Cerebral hemorrhage scores at 24 h after tPA treatment for MCAO/R (*n* = 6). (I) Quantitative analysis of cerebral hemorrhage volume at 24 h after tPA treatment for MCAO/R (*n* = 6). ^&&^ *p* < 0.01 and ^&&&^ *p* < 0.001 vs. Sham; ^#^ *p* < 0.05 and ^##^ *p* < 0.01 vs. MCAO/R; *p < 0.05 and **p < 0.01 vs. tPA + vehicle. Data are expressed as mean ± SD.

To evaluate the potential therapeutic effects of pachymic acid on ischemic stroke and the impact of tPA‐induced HT, we performed MCAO surgery on male C57BL/6J mice and administered tPA and pachymic acid simultaneously during reperfusion. Behavioral tests were conducted 24 h after reperfusion, and brains were harvested for relevant experiments (Figure 1A). TTC staining showed that, compared with the Sham group, the model group had obvious infarct areas (Figure 1B,D). Compared with the tPA+ vehicle group, both the tPA+ pachymic acid‐20 mg/kg and tPA+ pachymic acid‐40 mg/kg groups reduced the infarct volume in mice, with the most significant effect observed in the tPA+ pachymic acid‐80 mg/kg group (*p* < 0.01, Figure 1B,D).

In addition, neurosensory and motor functions were evaluated using mNSS, foot‐fault, and adhesive removal tests. Compared to the sham group, MCAO mice exhibited significantly impaired neurological functions, with increased mNSS scores (Figure 1E), elevated foot‐fault rates (Figure 1F), and prolonged adhesive removal times (Figure 1G). Although no statistical differences were observed between the MCAO/R and tPA + vehicle groups, trends toward worsening were noted (Figure 1E–G). Pachymic acid‐80 mg/kg treatment significantly reduced mNSS scores (*p* < 0.05, Figure 1E), foot‐fault rates (*p* < 0.05, Figure 1F), and adhesive removal times (Figure 1G), indicating pachymic acid's potential to improve neurological outcomes after tPA treatment.

Subsequently, we further investigated the potential efficacy of pachymic acid in mitigating tPA‐induced HT following cerebral ischemia. At 24 h post‐surgery, brains were harvested, and coronal sections were scored for intracerebral hemorrhage (Figure 1C,H). Hemoglobin content in the ischemic hemisphere was measured by spectrophotometry (Figure 1I). Compared with the sham group, the model group showed distinct infarct areas, confirming successful model establishment. Mice treated with tPA+ pachymic acid‐80 mg/kg exhibited significantly lower hemorrhage scores and volumes than those treated with tPA + vehicle (*p* < 0.05, Figure 1H,I), indicating a dose‐dependent trend. These results suggest that pachymic acid mitigates tPA‐induced HT following ischemic stroke. The mortality rates for the model group, tPA group, tPA+ pachymic acid‐20 mg/kg group, tPA+ pachymic acid‐40 mg/kg group, and tPA+ pachymic acid‐80 mg/kg group are 14%, 37.5%, 26.7%, 21.4%, and 20%, respectively.

### 3.2 Pachymic Acid Attenuates Tight Junction Protein Degradation and Protects BBB Integrity in tPA‐Treated MCAO/R Mice

**FIGURE 2:**
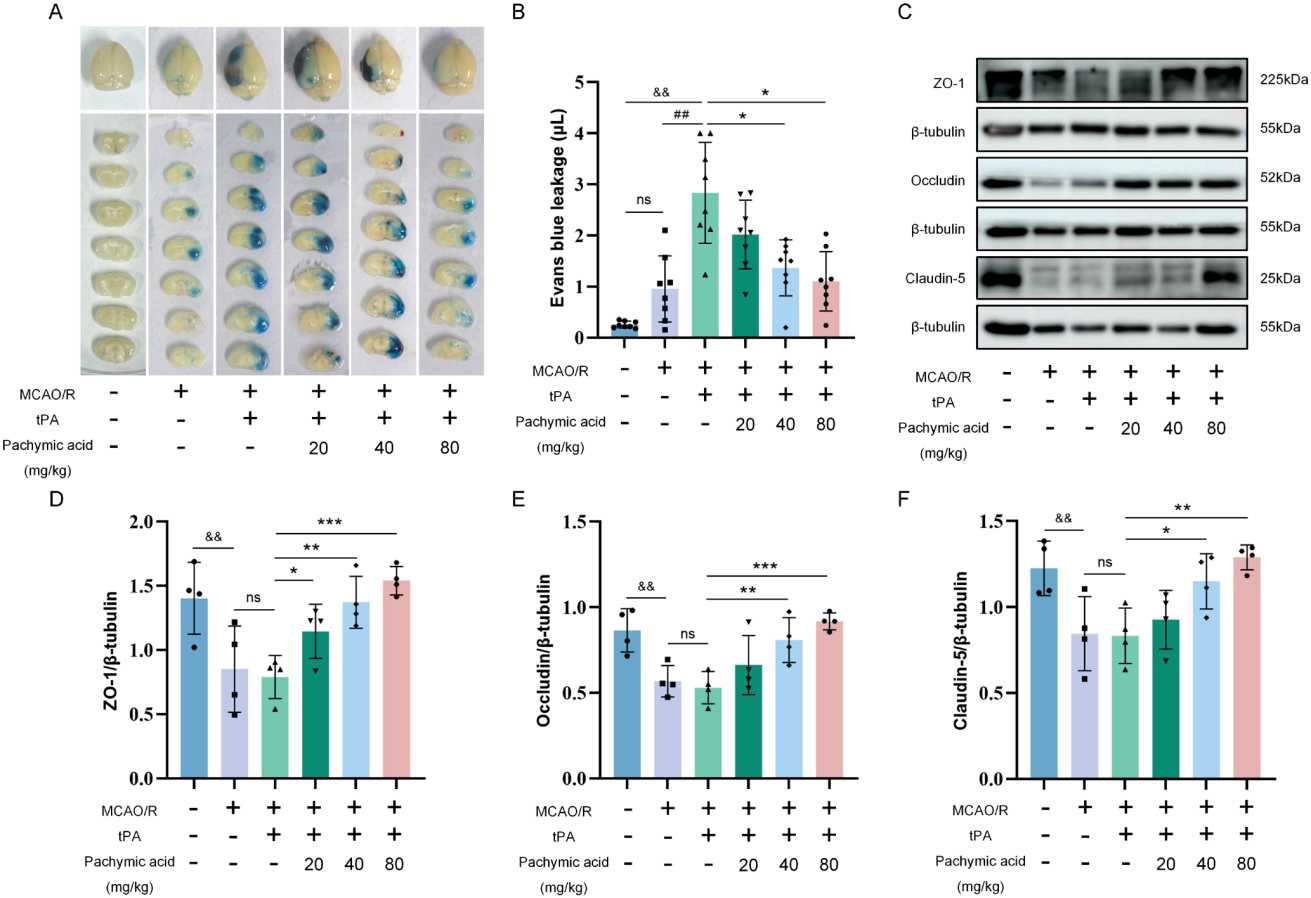
Pachymic acid attenuates BBB damage following tPA‐induced ischemic stroke. Representative images of brain top views and coronal sections at 24 h after tPA treatment for MCAO/R (A) and quantitative analysis (*n* = 8, B). (C) Representative expression of ZO‐1, Occludin, and Claudin‐5 proteins detected by Western blot. (D) Quantitative analysis of ZO‐1 protein (*n* = 4). (E) Quantitative analysis of Occludin protein (*n* = 4). (F) Quantitative analysis of Claudin‐5 protein (*n* = 4). ^&&^ *p* < 0.01 vs. Sham; ^##^ *p* < 0.01 vs. MCAO/R; *p < 0.05, **p < 0.01, and ***p < 0.001 vs. tPA + vehicle. Data are expressed as mean ± SD.

tPA‐induced HT is primarily caused by BBB disruption due to cerebral ischemia, which increases BBB permeability, leading to extravasation of blood and intracerebral hematoma formation, thereby exacerbating brain injury. Then, we used the Evans blue leakage assay to assess whether pachymic acid mitigates the tPA‐induced increase in BBB permeability. Results showed that, compared with the Sham group, MCAO/R mice exhibited significantly increased brain Evans blue content, which was further increased by intravenous tPA (*p* < 0.01, Figure 2A,B). Notably, treatment with pachymic acid (40 and 80 mg/kg) reduced the content of Evans blue in the brain, with a certain dose dependence (*p* < 0.05, Figure 2A,B).

Subsequently, we examined the effects of pachymic acid on the expression of tight junction proteins ZO‐1, Occludin, and Claudin‐5 (Figure 2C). Compared with the Sham group, MCAO/R significantly decreased the expression of ZO‐1, Occludin, and Claudin‐5 in the brain (*p* < 0.01), with further reductions following tPA treatment (Figure 2D–F). Compared with the tPA + vehicle group, pachymic acid (40 and 80 mg/kg) treatment increased the expression of ZO‐1, Occludin, and Claudin‐5, with significant effects observed at the pachymic acid‐80 mg/kg dose (*p* < 0.05, Figure 2D–F).

**FIGURE 3:**
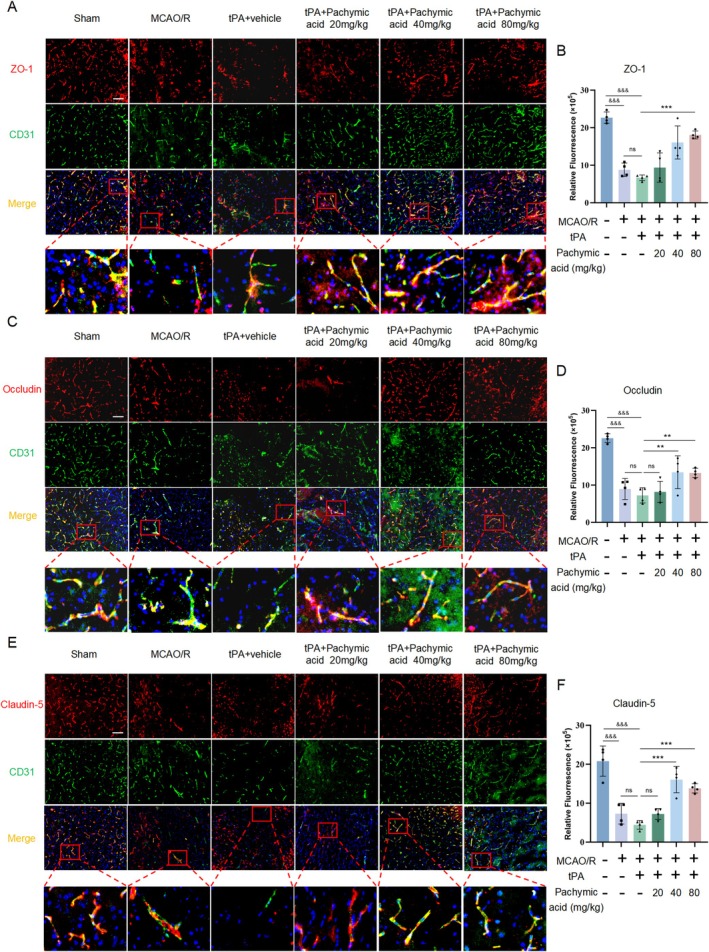
Pachymic acid increases ZO‐1, Occludin, and Claudin‐5 immunofluorescence intensity following tPA‐induced ischemic stroke. Representative expression of ZO‐1 protein detected by double immunofluorescence staining (A) and quantitative analysis (B). Representative expression of Occludin protein detected by double immunofluorescence staining (C) and quantitative analysis (D). Representative expression of ZO‐1 protein detected by double immunofluorescence staining (E) and quantitative analysis (F). Scale bar, 50 μm. ^&&&^ *p* < 0.001 vs. Sham; **p < 0.01 and ***p < 0.001 vs. tPA + vehicle. Data are expressed as mean ± SD.

To further demonstrate the protective effects of pachymic acid on the BBB, we performed CD31/ (ZO‐1, Occludin, or Claudin‐5) double immunofluorescence staining, and the results were consistent with those of WB. In the MCAO/R group, the fluorescence intensity of ZO‐1 (Figure 3A,B), Occludin (Figure 3C,D), and Claudin‐5 (Figure 3E,F) on brain microvascular endothelial cells was significantly decreased (*p* < 0.001). After tPA intervention, the fluorescence intensity of ZO‐1, Occludin, and Claudin‐5 was further decreased. In contrast, treatment with pachymic acid (40 and 80 mg/kg) significantly restored the fluorescence intensity of ZO‐1 (*p* < 0.001, tPA+ pachymic acid‐80 mg/kg vs. tPA+ vehicle), Occludin (*p* < 0.01, tPA+ pachymic acid‐80 mg/kg vs. tPA+ vehicle), and Claudin‐5 (*p* < 0.001, tPA+ pachymic acid‐80 mg/kg vs. tPA+ vehicle) in endothelial cells (Figure 3A–F).

**FIGURE 4:**
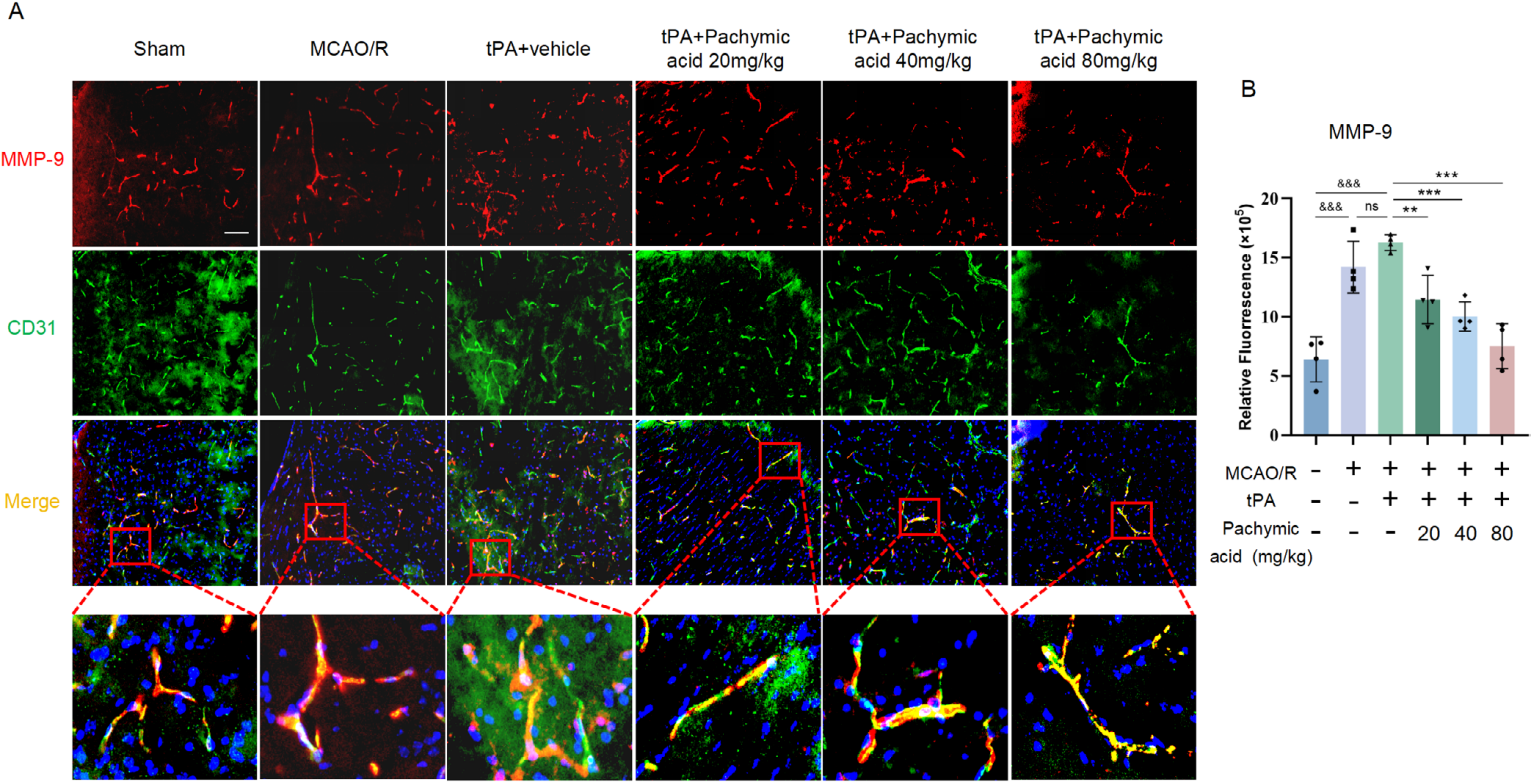
Pachymic acid reduces MMP‐9 immunofluorescence intensity following tPA‐induced ischemic stroke. Representative expression of MMP‐9 protein detected by double immunofluorescence staining (A) and quantitative analysis (B), scale bar, 50 μm. ^&&&^ *p* < 0.001 vs. Sham; **p < 0.01 and ***p < 0.001 vs. tPA + vehicle. Data are expressed as mean ± SD.

Given that excessive MMP‐9 activation is associated with BBB disruption and neuronal injury, we examined MMP‐9 expression in endothelial cells. Immunofluorescence revealed that compared to the sham group, MMP‐9 fluorescence intensity was significantly increased in the MCAO/R group (*p* < 0.001) and further increased by tPA treatment (Figure 4A,B). Notably, treatment with pachymic acid (20, 40, and 80 mg/kg) significantly reduced the fluorescence intensity of MMP‐9 (*p* < 0.001, tPA+ pachymic acid‐80 mg/kg vs. tPA+ vehicle, Figure 4A,B). These findings suggest that pachymic acid attenuates the degradation of tight junction proteins and reduces MMP‐9 expression induced by tPA treatment, thereby protecting BBB integrity.

### 3.3 Molecular Docking, Molecular Dynamics Simulations, and SPR Collectively Reveal the Direct Interaction of Pachymic Acid With the PI3K

Subsequently, we investigated the molecular mechanisms underlying pachymic acid's effects in MCAO/R lesions. Previous studies have demonstrated that pachymic acid exerts significant neuroprotective effects on brain I/R injury and neuronal apoptosis via the PI3K/Akt signaling pathway. Therefore, we hypothesize that pachymic acid may improve tPA‐induced HT following MCAO/R through the PI3K/Akt signaling pathway.

**FIGURE 5:**
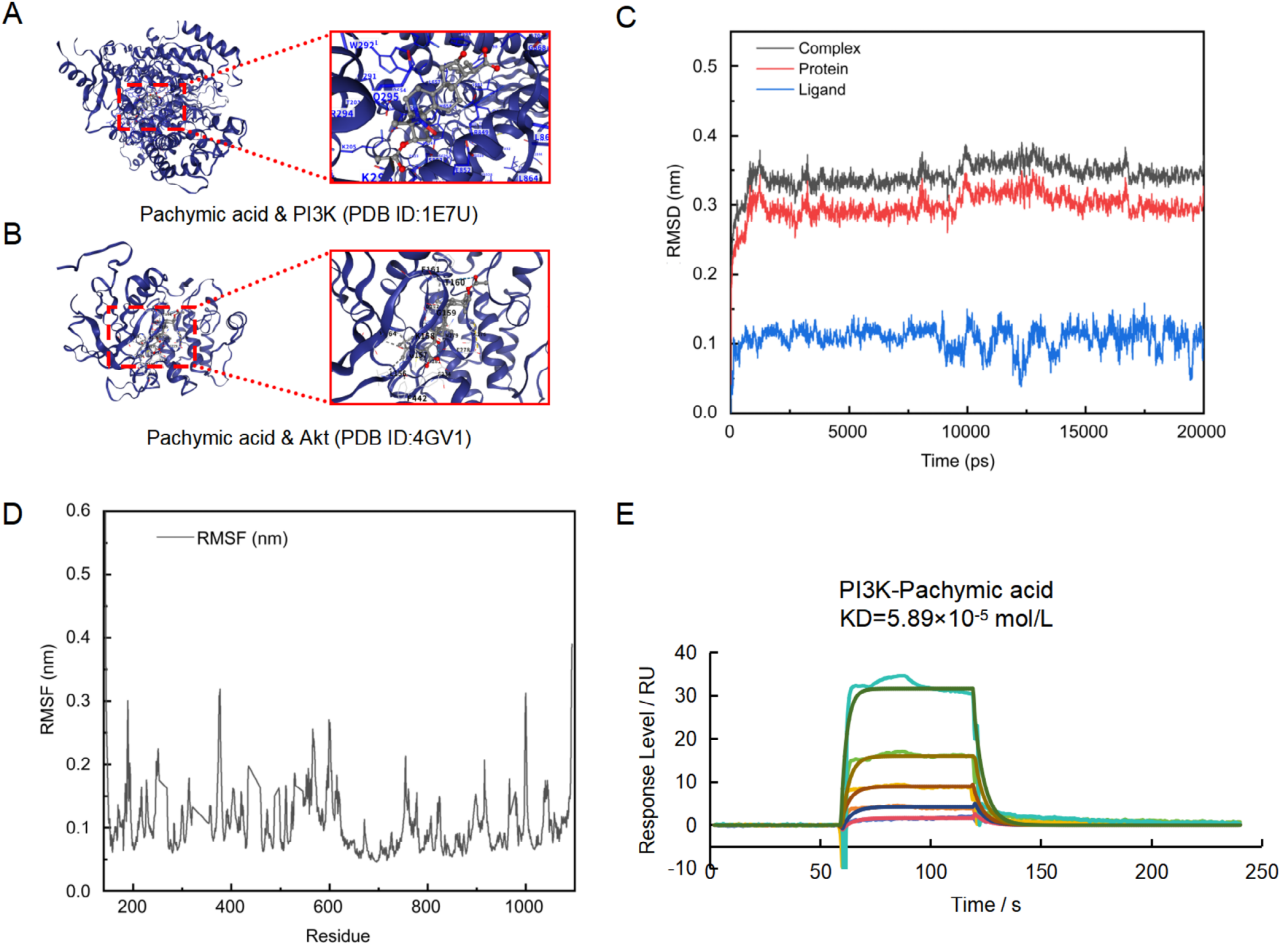
PI3K is a direct target of pachymic acid. (A) Molecular docking of pachymic acid with PI3K (PDB ID: 1E7U). (B) Molecular docking of pachymic acid with Akt (PDB ID: 4GV1). (C) RMSD of pachymic acid with PI3K. (D) RMSF of pachymic acid with PI3K. (E) SPR analysis of pachymic acid and PI3K interaction (KD = 5.89 × 10^−5^ mol/L).

Initially, we performed molecular docking of pachymic acid with the PI3K/Akt pathway using CB‐dock2 [22, 23] (Figure 5A,B). Upon docking pachymic acid with PI3K, a high score of −9.6 was obtained, compared with −8.3 for Akt, suggesting that PI3K may be the direct target of pachymic acid. To verify this, we used MD simulations and SPR to confirm the binding of pachymic acid to PI3K. The RMSD value, indicating system stability, stabilized below 0.4 nm around 1200 ps (Figure 5C), showing stable pachymic acid‐PI3K binding. As shown in Figure 5D, RMSF values for residues remained within 0.2 nm, with no significant fluctuations. Key residues in the binding pocket exhibited consistently low RMSF values, indicating stable interactions between pachymic acid and the binding pocket. Further, we used SPR to detect the direct interaction between PI3K and pachymic acid. As shown in Figure 5E, PI3K and pachymic acid exhibited specific binding with a dissociation constant (KD) of 5.89 × 10^−5^ mol/L, confirming that PI3K is a direct target of pachymic acid.

### 3.4 Pachymic Acid Activates the PI3K/Akt Signaling Pathway to Reduce the Degradation of Tight Junction Proteins in MCAO/R Mice After tPA Treatment

**FIGURE 6:**
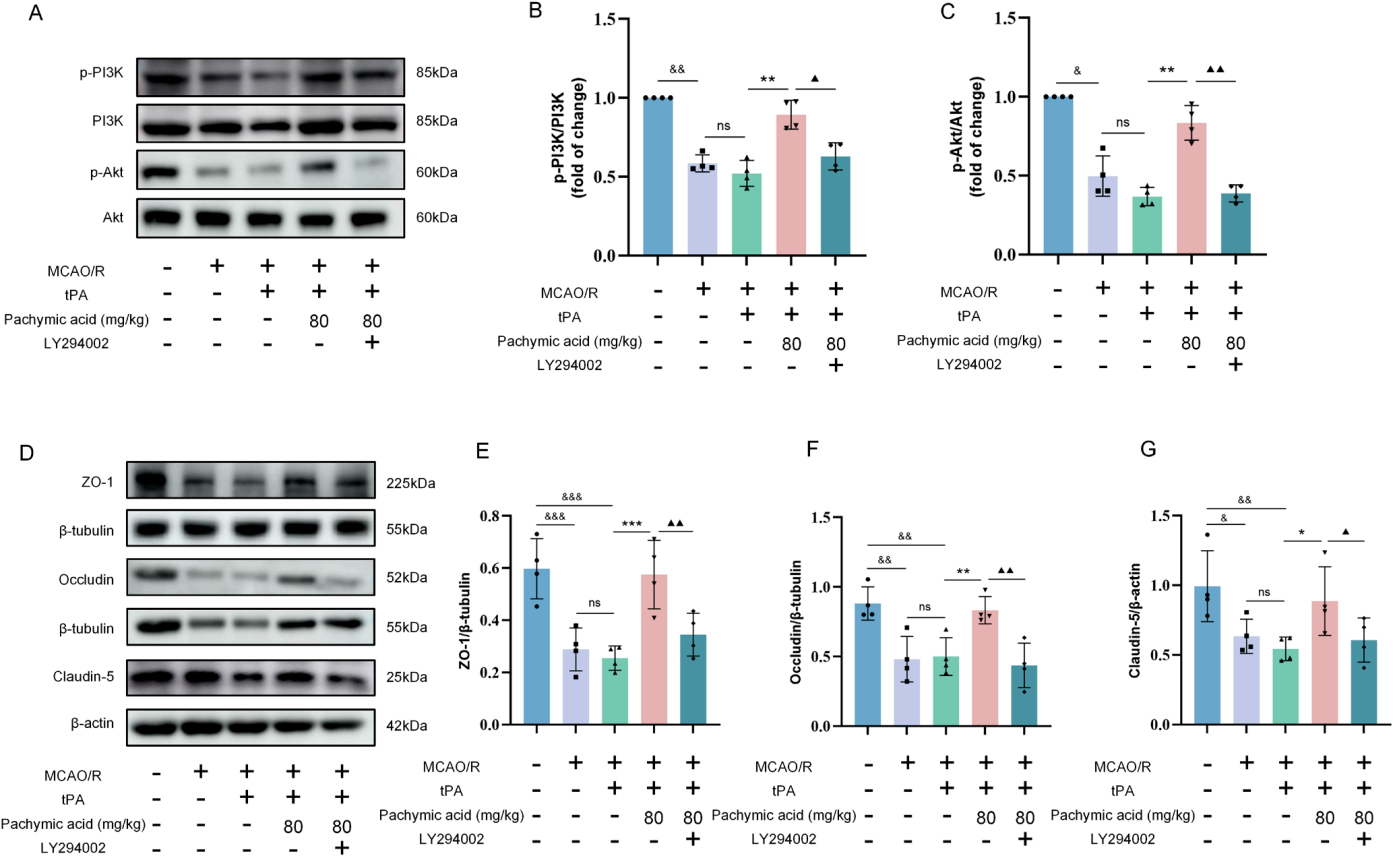
Pachymic acid reduces tight junction protein degradation via the PI3K/Akt pathway. Representative expression of p‐PI3K, PI3K, p‐Akt, and Akt proteins detected by Western blot (A) and quantitative analysis (B‐C, *n* = 4). (D) Representative expression of ZO‐1, Occludin, and Claudin‐5 proteins detected by Western blot (*n* = 4). (E) Quantitative analysis of ZO‐1 protein (*n* = 4). (F) Quantitative analysis of Occludin protein (*n* = 4). (G) Quantitative analysis of Claudin‐5 protein (*n* = 4). ^&^ *p* < 0.05, and ^&&^ *p* < 0.01 and ^&&&^ *p* < 0.001 vs. Sham; **p < 0.01 vs. tPA + vehicle; ^▲^ *p* < 0.05 and ^▲▲^ *p* < 0.01 vs. tPA + pachymic acid‐80 mg/kg. Data are expressed as mean ± SD.

We further validated the role of the PI3K/Akt signaling pathway in in vivo experiments by injecting the PI3K inhibitor LY294002 into the lateral ventricle of C57BL/6J mice before MCAO/R surgery to observe the effects of pachymic acid on tPA‐induced HT damage (Figure 6A). Western blot analysis revealed that compared with the Sham group, the expression of p‐PI3K and p‐Akt was significantly reduced in the MCAO/R groups and showed a trend of further reduction after tPA treatment (Figure 6B,C). Compared with the tPA+ vehicle group, the tPA+ pachymic acid‐80 mg/kg group promoted the expression of p‐PI3K and p‐Akt (*p* < 0.01, Figure 6B,C). However, when the PI3K/Akt signaling pathway was inhibited by LY294002, the promoting effects of pachymic acid on p‐PI3K (*p* < 0.05) and p‐Akt (*p* < 0.01) expression were reversed (Figure 6B,C).

To verify the effects of pachymic acid‐activated PI3K/Akt signaling on tight junction expression in the BBB, we examined the expression of tight junction proteins following inhibition of the PI3K/Akt pathway by LY294002 (Figure 6D). Western blot analysis revealed that, compared with the Sham group, the expression of ZO‐1 (Figure 6E), Occludin (Figure 6F), and Claudin‐5 (Figure 6G) significantly decreased after MCAO/R, with further reductions observed following tPA treatment. However, pachymic acid treatment attenuated the degradation of tight junction proteins. Notably, the protective effects of pachymic acid on tight junction proteins were abolished by LY294002 treatment (Figure 6E–G). These findings suggest that pachymic acid protects tight junction proteins in the BBB, thereby alleviating HT by activating the PI3K/Akt signaling pathway during tPA‐induced HT.

### 3.5 Pachymic Acid Attenuates tPA‐Induced HT and Improves Prognosis in MCAO/R Mice by Activating the PI3K/Akt Signaling Pathway

**FIGURE 7:**
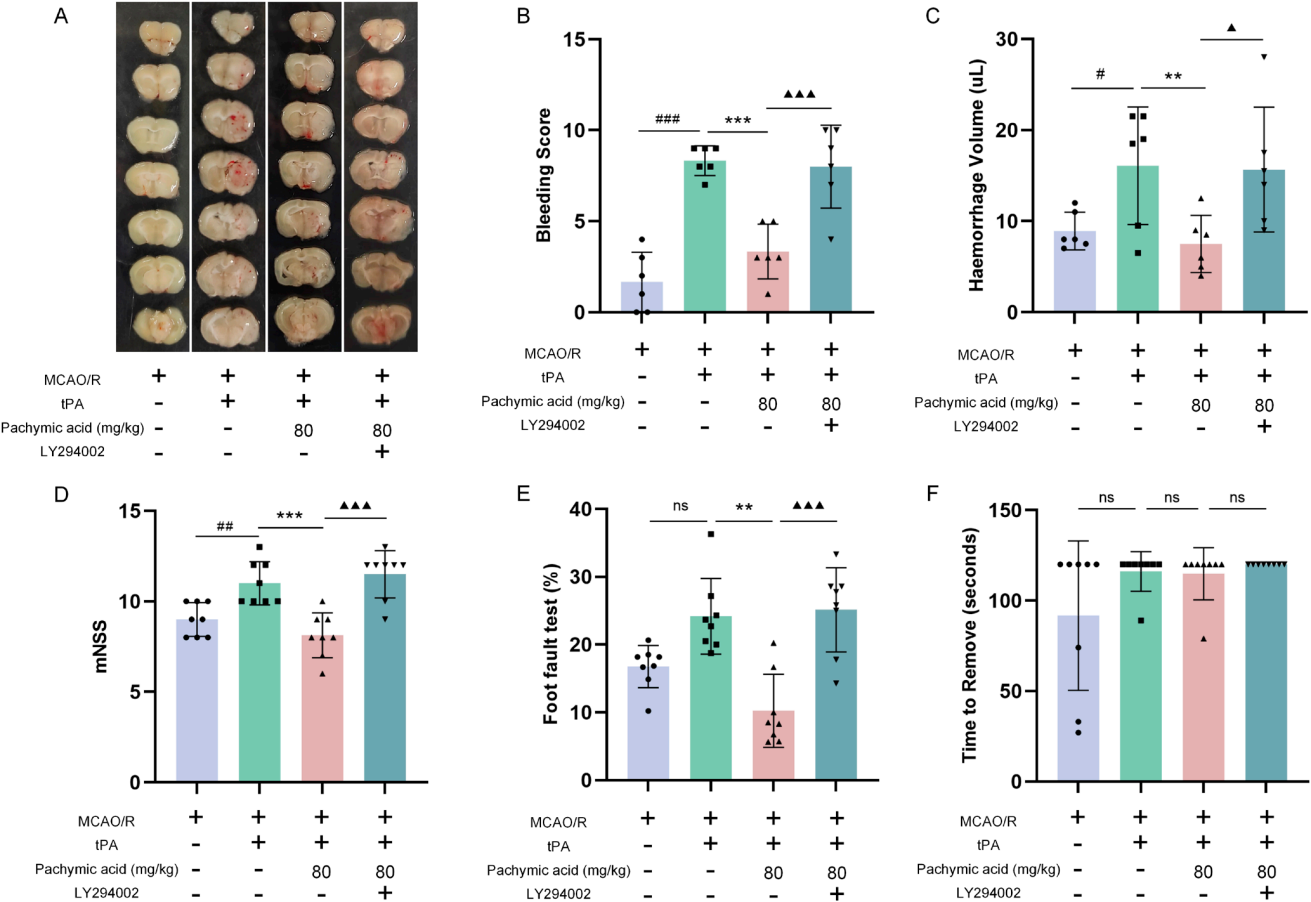
LY294002 abrogates the effects of pachymic acid on reducing tPA‐induced HT and improving MCAO/R outcomes. (A) Representative coronal brain sections at 24 h after tPA treatment for ischemic stroke. (B) Cerebral hemorrhage scores (*n* = 6). (C) Quantitative analysis of cerebral hemorrhage volume (*n* = 6). Effects of pachymic acid on behavioral deficits in MCAO/R mice: mNSS test (*n* = 8, D), foot‐fault test (*n* = 8, E), and adhesive removal test (*n* = 8, F). ^#^ *p* < 0.05, ^##^ *p* < 0.01, and ^###^ *p* < 0.001 vs. MCAO/R; **p < 0.01 and ***p < 0.001 vs. tPA + vehicle; ^▲^ *p* < 0.05 and ^▲▲▲^ *p* < 0.001 vs. tPA+ pachymic acid‐80 mg/kg. Data are expressed as mean ± SD.

Subsequently, we further explored the effects of pachymic acid on tPA‐induced HT and neurological function following ischemic stroke after inhibiting the PI3K/Akt signaling pathway. Consistent with previous results, compared with the MCAO/R group, intravenous tPA administration 1 h after cerebral ischemia increased the risk of HT, as evidenced by increased cerebral hemorrhage volume and significant elevation in hemorrhage scores (Figure 7A–C). Pachymic acid treatment attenuated tPA‐induced HT, with the tPA+ pachymic acid‐80 mg/kg group showing significantly lower hemorrhage scores than the tPA+ vehicle group (*p* < 0.001). Notably, compared with the tPA+ pachymic acid‐80 mg/kg group, the PI3K inhibitor group exhibited higher hemorrhage scores (*p* < 0.001) and cerebral hemorrhage volume (*p* < 0.05) (Figure 7B,C). Neurological tests revealed that tPA exacerbated neurological deficits following MCAO/R (*p* < 0.01, Figure 7D–F). Compared with the tPA+ vehicle group, pachymic acid treatment improved mNSS (*p* < 0.001, Figure 7D) and reduced foot‐fault rate (*p* < 0.01, Figure 7E). However, these effects were reversed by LY294002 (*p* < 0.001, Figure 7D,E). No significant differences were observed in the time taken to remove adhesive tape among groups (Figure 7F). These experiments further demonstrated that the effects of pachymic acid on tPA‐induced HT and neurological repair in MCAO/R mice are mediated through the PI3K pathway.

## 4 Discussion

Ischemic stroke is one of the major diseases that severely threaten human health and life [24]. The only thrombolytic drug currently used in the clinic for acute ischemic stroke is tPA. However, the narrow therapeutic time window and severe HT greatly limit its clinical use [8], and researchers have been actively seeking new strategies to reduce the risk of HT and improve the thrombolytic efficacy of tPA. It is widely believed that tPA‐induced HT is mainly caused by inflammation and further disruption of the BBB following reperfusion. Increased BBB permeability allows harmful macromolecules to infiltrate, disrupting central nervous system homeostasis, exacerbating cerebral edema and infarction, and worsening brain I/R injury. Additionally, cerebral ischemia significantly increases MMP‐9 expression, which degrades the extracellular matrix and tight junction proteins, compromising BBB integrity and causing cerebral edema, inflammatory cell infiltration, and HT [25]. Therefore, targeting BBB tight junction repair is crucial for alleviating brain I/R injury. Pachymic acid, a triterpenoid compound from *Poria cocos*, has anti‐inflammatory and antioxidant effects and reduces BBB disruption in mice post‐ischemia. However, the effects and molecular mechanisms of pachymic acid in reducing tPA‐induced HT have not been reported.

In this study, we established a focal cerebral ischemia model in mice and administered tPA via the femoral vein during reperfusion at 1 h after ischemia. This model mimics acute ischemic stroke in the clinic and allows for tPA reperfusion treatment within a defined therapeutic time window. In the current study, administration of tPA following MCAO surgery did not increase cerebral infarct volume or worsen neurological outcomes in mice. This may be attributed to the fact that at 24 h post‐stroke, animals remain in the acute phase of severe injury, where neurological scores (such as mNSS) may have approached a “floor effect,” thus limiting the ability to detect additional impairment specifically attributable to HT. Concurrently, the long‐term effects of hemorrhage had not yet fully manifested. However, it is noteworthy that tPA treatment after MCAO significantly increased HT, as evidenced by higher hemorrhage scores and elevated cerebral hemoglobin content in the ischemic hemisphere. Following pachymic acid treatment, mice exhibited improvements in infarct volume, neurological outcome, hemorrhage scores, and hemoglobin content. These findings suggest that pachymic acid may enhance the safety of tPA use. However, the mechanisms underlying pachymic acid's protective effects against tPA‐induced HT remain unexplored.

Given that reperfusion therapy for ischemic stroke further disrupts the BBB, we hypothesize that pachymic acid prevents tPA‐induced HT by protecting BBB tight junction proteins. Our study shows that BBB disruption is more severe in tPA‐treated mice following MCAO/R, as evidenced by increased Evans blue leakage and significant downregulation of tight junction proteins, such as ZO‐1, Occludin, and Claudin‐5. In contrast, pachymic acid treatment significantly restored the expression of these proteins. Immunofluorescence staining revealed that MMP‐9 expression was upregulated in mice subjected to MCAO/R and tPA treatment but downregulated following pachymic acid treatment. Collectively, these results indicate that pachymic acid reduces MMP‐9 expression, mitigates BBB damage, and decreases the degradation of tight junction proteins, thereby protecting BBB integrity and improving tPA‐induced HT following MCAO/R.

The PI3K/Akt signaling pathway is a survival pathway associated with brain I/R injury. It exerts anti‐inflammatory, antioxidant, and BBB‐protective effects [7], and plays an intricate role in tPA‐induced BBB damage [6]. Previous studies have reported that pachymic acid exerts significant neuroprotective effects on brain I/R injury and neuronal apoptosis [10]. However, the precise role of pachymic acid in the PI3K/Akt pathway in improving tPA‐induced HT remains unclear. Notably, our molecular docking results indicate a strong binding affinity between pachymic acid and PI3K (−9.6 kcal·mol^−1^), suggesting PI3K as a potential direct target of pachymic acid. Subsequent MD simulations and SPR experiments further confirmed that pachymic acid can directly bind to PI3K protein and that the binding is stable. Based on these findings, we hypothesize that pachymic acid protects the BBB by directly binding to PI3K and activating the PI3K/Akt pathway. To investigate this interaction, we administered the PI3K‐specific inhibitor LY294002 into the lateral ventricle of mice. The results showed that pachymic acid treatment restored the decreased expression of p‐PI3K and p‐Akt following tPA treatment, an effect inhibited by LY294002. These findings suggest that pachymic acid alleviates cerebral ischemic injury by activating the PI3K/Akt pathway. Further experiments revealed that LY294002 abolished pachymic acid's protective effects against tPA‐induced HT and its neuroprotective effects following ischemic stroke. These results indicate that pachymic acid reduces tight junction protein degradation and mitigates BBB disruption by binding to PI3K, thereby activating the PI3K/Akt pathway.

Notably, tPA‐induced HT arises from a complex interplay of multiple mechanisms. While our investigation centers on the PI3K/Akt pathway, the critical role of oxidative/nitrosative stress in reperfusion injury following delayed thrombolysis [26] must be acknowledged. Cerebral ischemia–reperfusion instigates a burst of reactive oxygen and nitrogen species (ROS/RNS), whose reaction yields the potent oxidant, peroxynitrite [27]. This agent serves as a pivotal upstream activator of MMP‐9 [28], both directly cleaving its zymogen and inducing its transcription via factors [29] such as NF‐κB, thereby precipitating a surge in MMP‐9 activity. Consequently, activated MMP‐9 degrades key constituents of the BBB—the basement membrane and tight junction proteins—leading to structural failure and HT [30, 31]. Concurrently, pachymic acid has been documented to exhibit significant antioxidant effects [32, 33]. Therefore, elucidating the relationship between the HT reduction by pachymic acid via the PI3K/Akt pathway and the ROS/RNS‐MMP‐9 axis has become a core scientific question that must be addressed.

Studies have established that p‐Akt serves as a key upstream regulator of MMP‐9 expression. This occurs primarily through the Akt/NF‐κB signaling axis: activated Akt phosphorylates and activates IκB kinase (IKK), leading to the degradation of IκB protein. This process releases the transcription factor NF‐κB, allowing it to translocate into the nucleus. NF‐κB can directly bind to the MMP‐9 promoter and potently drive its transcription [34]. Therefore, according to canonical theory, Akt activation would be expected to upregulate MMP‐9 expression. However, the current study demonstrates that pachymic acid activates the PI3K/Akt pathway while simultaneously significantly reducing cerebral MMP‐9 expression after tPA treatment, alleviating HT. Furthermore, the protective effects of pachymic acid were reversed upon inhibition of the PI3K/Akt pathway with LY294002. This appears to contradict the established understanding that “Akt activation can upregulate MMP‐9 expression via NF‐κB”. This seemingly paradoxical finding leads us to propose an integrative hypothesis: Within the specific pathological context of tPA‐induced HT, the primary function of the PI3K/Akt signal activated by pachymic acid may not be to drive MMP‐9 expression, but rather to act as a core “synergistic stabilizer”. A potential mode of action is that pachymic acid first suppresses the ROS/RNS‐MMP‐9 axis via other pathways (potentially including its inherent antioxidant properties), thereby blocking the excessive generation and activation of MMP‐9. Concurrently, the PI3K/Akt pathway, which pachymic acid activates, focuses on stabilizing and repairing the tight junction structure of the BBB by phosphorylating downstream substrates. These two pathways act in concert to jointly defend against HT.

A key limitation is that the current study did not directly measure cerebral ROS/RNS or related oxidative stress markers. Thus, the study lacks direct evidence for pachymic acid's intervention on the ROS/RNS‐MMP‐9 axis, though the integrative hypothesis offers a logical explanation. Future research will directly measure oxidative stress markers, such as peroxynitrite and 3‐nitrotyrosine, to empirically demonstrate pachymic acid's upstream inhibitory effect on this axis and to further elucidate its role in regulating the relationship between the ROS/RNS‐MMP‐9 axis and the PI3K/Akt pathway.

Additionally, the current study has other limitations. (1) While PI3K/Akt is necessary for protection, its cell‐type‐specific roles within the neurovascular unit (e.g., endothelial cells, neurons, astrocytes) are unclear. Future studies utilizing cell‐specific knockout models or primary cell co‐culture systems will be essential to precisely identify the cellular targets of pachymic acid. (2) The functional assessments were primarily focused on the 24‐h post‐reperfusion time point, which represents the peak period for HT. Future studies should include earlier (6, 12 h) and later (3, 7d) time points to better define the dynamic profile of BBB disruption and treatment. (3) Pachymic acid concentrations in the brain were not measured, which are essential for definitive pharmacokinetic characterization. Subsequent experiments should include reporting the brain concentration of pachymic acid post‐administration. (4) The study's focus on young healthy male mice, which limits clinical extrapolation. Future study must validate the strategy in more clinically relevant models (aged, female) and test its efficacy with delayed tPA administration (2–3 h or > 4.5 h) to address clinical translatability.

## 5 Conclusion

In summary, the current study demonstrates that pachymic acid can directly bind to PI3K, thereby activating the PI3K/Akt signaling pathway, protecting the BBB, and alleviating HT induced by tPA after ischemic stroke. In short, pachymic acid holds promise as a potential therapeutic agent for ongoing ischemic stroke research, offering the possibility of reducing HT, which is induced by tPA during reperfusion after ischemic stroke.

## Author Contributions

**Yongshi Wu:** investigation, formal analysis, writing – original draft. **Tong Zhang:** investigation, methodology, writing – review and editing. **Ruoqi Li:** investigation, methodology. **Yige Wu:** investigation. **Congmin Wei:** formal analysis. **Fangming Sun:** formal analysis. **Shanshan Zhang:** supervision, writing – review and editing. **Xiang Fan:** conceptualization, funding acquisition, Supervision, writing – review and editing. All authors read and approved the final manuscript. All data were generated in‐house. All authors agree to take responsibility for all aspects of the work to ensure completeness and accuracy.

## Funding

This work was supported by the Zhejiang Provincial Natural Science Foundation of China, LY22H280004.

## Ethics Statement

All animal experimental procedures were approved by the Committee for the Use and Protection of Laboratory Animals of Zhejiang Chinese Medical University (Ethics No. 20230515–19) and followed the Guidelines for the Care and Use of Laboratory Animals.

## Conflicts of Interest

The authors declare no conflicts of interest.

## Data Availability

The data that support the findings of this study are available from the corresponding author upon reasonable request.
